# A qualitative study of lived experience and life courses following dam release flooding in Northern Ghanaian communities: Implications for damage and loss assessment

**DOI:** 10.1371/journal.pone.0310952

**Published:** 2024-12-02

**Authors:** Moses Asamoah, Mawuli Dzodzomenyo, Faustina Twumwaa Gyimah, Chengxiu Li, Linda Agyemang, Jim Wright

**Affiliations:** 1 Ghana School of Public Health, University of Ghana, Legon, Accra, Ghana; 2 School of Geography and Environmental Science, University of Southampton, Highfield, Southampton, United Kingdom; 3 Institute of Statistical, Social and Economic Research, University of Ghana, Legon, Accra, Ghana; 4 Department of Nursing Science, University of Bournemouth, Fern Barrow, United Kingdom; University of Sargodha, PAKISTAN

## Abstract

**Background:**

Dams provide water for industrial, agricultural, and domestic use, particularly in arid regions. However, controlled dam releases due to heavy rainfall may affect downstream communities’ livelihoods and life courses such long-term impacts may be omitted from damage and loss assessments. This study aims to assess the lived experiences and long-term consequences of dam release flooding for downstream populations, comparing these with the typical scope of a damage and loss assessment (DaLA).

**Methods:**

This research was conducted in two flood-prone districts in the White Volta basin, Ghana, subject to dam spillage. Four Focus Group Discussions (FGDs) with community opinion headers, household heads, chiefs, local politicians, and institutional staff were conducted and analysed, alongside semi-structured interviews with twelve opinion leaders and disaster-related institutions.

**Results:**

Flood-affected communities struggled to attract partners for marriage due to stigmatisation from flooding impacts. Women outside flooded areas rejected male members’ marriage proposals, while communities offered young girls for marriage to wealthy men for greater financial security. Out-migration of female members to seek better livelihoods frequently led to divorce, subsequently affecting children’s education and well-being. Participants reported long-term trauma from flood-related contact with dangerous wildlife, travel disruption, disease risk, livelihood loss, and accidents. Such life course events and long-term trauma would be omitted from a DaLA exercise.

**Conclusion:**

Beyond its immediate impacts, flooding undermines family relationships and marriage, impairing children’s education and traumatises affected communities. We recommend livelihood diversification programmes, psychological support and family counselling to address these long-term impacts, with expansion of DaLA’s scope to underpin such support.

## Introduction

Together with storms, floods were the most frequent form of natural disaster globally in 2017 [1]. An estimated 1.81 billion people are exposed to 1-in-100 year floods, 89% of whom live in low and middle income countries. Of the 170 million facing both high flood risk and extreme poverty, 44% live in Sub-Saharan Africa [2]. Most floods in developing countries and tropical regions simultaneously affect the environment, agriculture, water resources, life course, and public health [3], with impacts exacerbated by poverty. The number of displaced people is often large, and deaths are also high [4], significantly impacting life course events.

Life course is described as "a sequence of socially defined events and roles that the individual enacts over time" [5]. Flooding has been found to shape later life course events such as marriage, children’s education and malnutrition. A longitudinal study by Ahmed [3] in Bangladesh revealed that flood years were associated with reduced marriage rates. Floods delayed or disrupted planned marriages. A focus group discussion in the Philippines revealed that repeated floods disrupted children’s education [6]. Focus group discussions and key informant interviews in the Muzarabani District of Zimbabwe showed that flood exposure subsequently reduced learning hours, qualified teaching staff, and syllabus coverage whilst increasing absenteeism, resulting in poor academic performance for children [7]. Flood exposure was related to long-term malnutrition in rural populations in the Indian state of Orissa. A household survey in flooded villages in Bangladesh also revealed that children exposed to flooding during their first year of life had a greater subsequent prevalence of chronic malnutrition [8].

Furthermore, some studies have revealed how flood events affect mental health. During the 2007 flood recovery across England, relocation was associated with psychological distress and a sixfold increase in mental health symptoms [9]. Few of these existing studies of flooding impacts on the life course focused on Sub-Saharan Africa, despite this being the most flood-susceptible world region [10].

Although the literature highlights a myriad of long term impacts of flooding on life course, techniques that have been developed to assess impact mostly focus on economic assets. According to a recent systematic review, the Damage and Loss Assessment (DaLA) framework is the most widely used approach to assessing natural disaster impacts [11]. DaLA involves the assessment of damage to infrastructure, the social sector (housing, health, and education), and productive sectors (agriculture, industry, commerce and tourism) [12]. DaLA is often used to determine damage impacts and loss following natural hazards to estimate government intervention for short-term and post-disaster financing needs [12]. DaLA has been criticised for focusing on the tangible and direct impacts of natural disasters at the expense of long-term and less tangible impacts [11]. It rarely considers the long-term aftermath impacts of life course transition such as marriage, lifestyle changes [3, 13]. psychological issues [9], divorce, childbirth, and child-wellbeing [8], social disruption and its impacts on children’s education [6, 7] and longer-term social capital, and migration in/out of flood-prone areas. Thus, the lived experience of flood-affected communities may not be fully reflected in the DaLA process, leading to a mismatch between community priorities and those emerging from DaLA assessments.

Dam-release flooding remains a specific form of flooding that is under-studied. Approximately 472 million people globally live downstream of large dams whose livelihoods depend on these dams [14]. Dam attenuation effects have been reported to reduce total flood susceptibility by 9%, protecting 590 million people globally from flood exposure [15]. However, in some cases, unexpected intense precipitation events in dam catchment areas have increased water inflows into large dams beyond planned capacity, leading to frequent and rapid spills to avoid dam wall collapse [16]. With climate change, extreme precipitation is expected to increase, and this amplification may further increase surface water runoff and, thereby, the risk of dam failure and spillage [17–21]. Understanding the role of dams in climate impact studies has become essential, as there were 37,600 dams higher than 15m worldwide in 2014, with over 3,700 large dams planned or under construction globally [15]. According to global modelling studies, dams ameliorate flood risk, but locally, they can increase flooding intensity and frequency [22]. Dam releases from spills usually cause flooding downstream [14] but their impacts on livelihoods are context-dependent. More specific evidence from local case studies on livelihood impacts is thus important to inform dam design and management sensitive to specific contexts.

Given this context, this study aims to understand the scope of dam release flooding impacts reported by downstream communities and key informants in relation to the range of impacts that would typically be covered by the DaLA framework. In doing so, it seeks to identify those impacts that are not captured through DaLA and particularly those affecting the life course, but which are a central part of the lived experience of affected communities. It was conducted in the White Volta catchment in Ghana among communities subject to frequent dam release flooding.

## Methods

### Case study area: The White Volta catchment, Ghana

**Fig 1:**
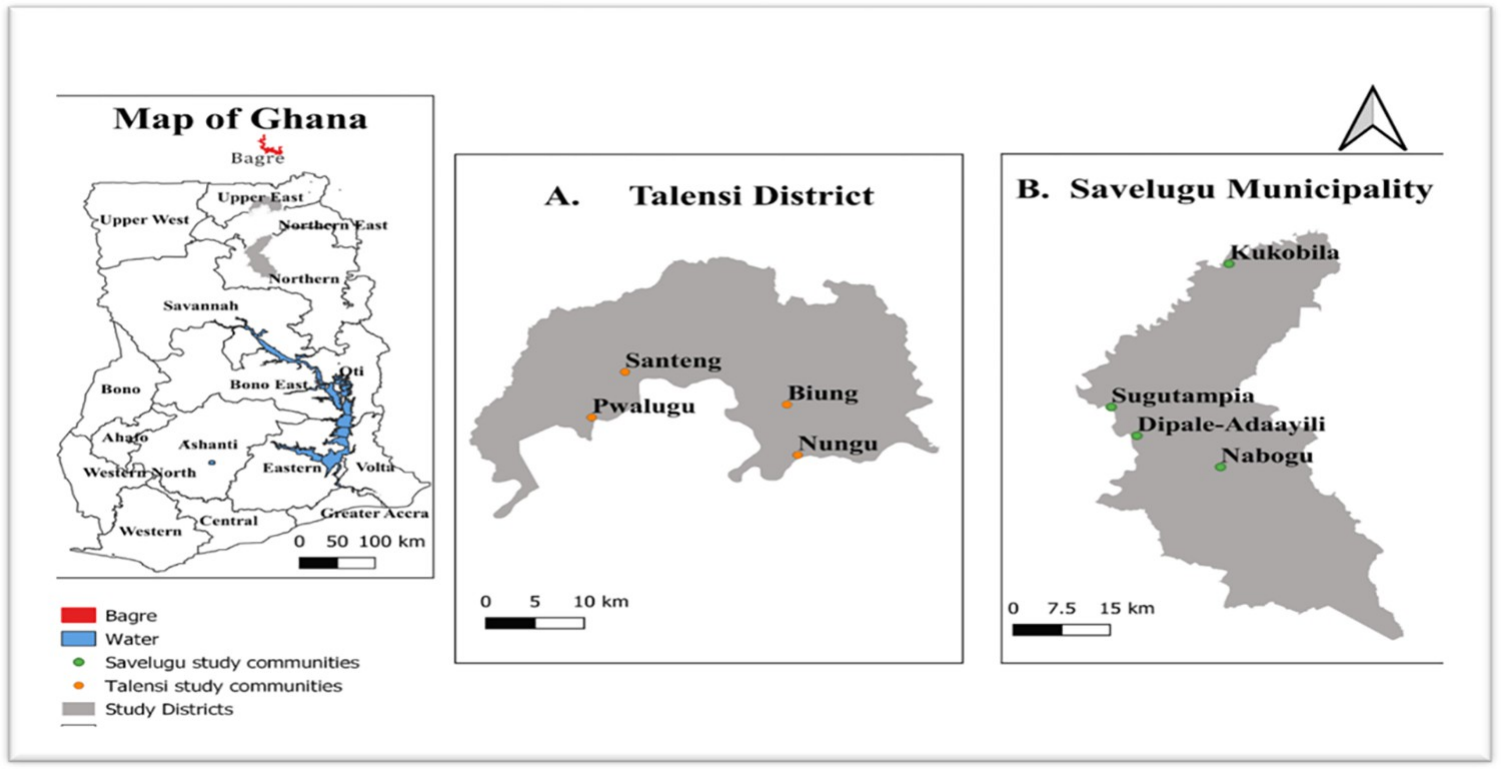
Map of study sites for focus group discussions and key informant interviews in Talensi and Savelugu Municipal Districts, Ghana. Note: This map was produced by the authors with GPS coordinates of the study sites collected during fieldwork and administrative boundaries for Ghana’s districts data from geoBoundaries [74].

Talensi District (Fig 1A) in Upper East Region, Ghana, had a total population of 87,021 in 2021 of which 88.2 per cent is rural [23]. The main source of employment is crop agriculture, through which about 90% of the population attain their livelihood. Livestock rearing, food manufacturing, firewood extraction, poultry production, logging, and tourism are other practices that people engage in. The secondary sector is small, comprising a cotton ginnery, a tomato factory, and two quarries. Savelugu Municipality (Fig 1B) in Northern Region had a population of 122,888 in 2021, of which 37.1% were rural. Its economic base is agriculture (mostly subsistence, smallholder farming), employing 74.1% of the economically active population. The White Volta passes through the municipality, allowing the population to fish and farm on its floodplain and serving as its primary water source. Both areas experience a unimodal rainfall regime from late April to mid-October, with average annual precipitation of 1000-1200mm.

These communities also suffer the most during dam release-related flooding. The Bagre dam is operated by a Burkinabe power company, Société Nationale Burkinabe d’Electricité (SONABEL) and has two turbines with a total installed capacity of 16MW. It meets 10% of the country’s energy supplies. The dam flows into the White Volta and enters Ghana from the village of Sapielga in the Upper East Region, about 60 kilometres from the dam [24]. This flooding regime began in the 1990s when the Bagre Dam could no longer retain water above its 235m depth during the rainy season [25]. The dam has had to spill water for the last two decades to prevent dam wall collapse. Due to the volume of inflows into the dam catchment area, this spillage normally occurs in August and September during the rainy season [26]. Relief agencies have reported adverse impacts from dam-related flooding in more than 265 communities in five regions of northern Ghana [27].

According to the joint rapid flood assessment in 2018 and 2019 conducted by the National Disaster Management Organization (NADMO) and the Ghana Red Cross Society (GRCS), a total of 3,556 and 5,010 households, respectively (21,336 and 26,083 people) were affected, out of which 6 (19 injured) and 21 (19 injured) deaths were confirmed in the Upper East Region in the respective years. A total of 487 and 2218 completely damaged houses and 7757 and 3743 partially damaged houses were recorded in these same years, respectively. These displaced inhabitants were sheltered in local schools, churches, local council buildings, and with relatives. Apart from these joint reports, there is no DaLA for the White Volta catchment. However, Dzodzomenyo et al. [28]. have assessed the flooding impact on microbiological contamination of domestic water sources, Guribie [29] has also assessed agricultural fields damages using Sentinel-1 SAR images and digital elevation models, and Nsor [30] have quantified floodplain vegetational change along the Volta river catchment in Northern Ghana.

### Study design

This study formed a component of a wider project, examining the impacts of flooding on livelihoods, the life course, healthcare utilisation [31], and the microbiological quality of water points [28, 32]. This project component employed a sequential exploratory approach through a mixed-methods study design [33]. It also used a hermeneutic phenomenological approach, as Van Manen [34] used to study lived experience [35]. This initial exploratory qualitative research phase of the larger mixed methods study aims to understand the human experience from the perspective of those who have lived it [36]. In studying the life course, the phenomenological approach focuses on how individuals make meaning of their experiences and how these experiences shape their development over time. De Brasi et al. [37], Smith et al. [38], Hareven [39] and Hutsebaut et al. [40] used a qualitative phenomenological approach to understand better how human lived experience in an event affects their life course.

This study uses this approach to understand the occurrence and severity of dam release flooding and its implications on life course and livelihoods from the perspectives of flood-affected communities and organisations engaged in flood preparedness/response via Focus Group Discussions (FGD) and Key Informant Interviews KIIs respectively [41].

### Sampling and area selection

Four communities were selected from Savelugu Municipal and Talensi districts using preliminary analysis of satellite imagery, corroborated by Red Cross reports documenting the agency’s emergency response to flooding in the Upper East region [42]. For these districts, satellite imagery from the Sentinel-1, Sentinel-2, and Landsat-8 sensors was acquired for known periods of Bagre Dam spillage (usually August-September) from 2015 to 2019. This imagery was classified to map the historical distribution of flooding following dam release using a differencing image technique [43, 44]. Study sites were identified using a composite inundation map layer created from the map layers of flooded areas for different years. This composite flood map was overlaid onto digital map layers of populated places based on the WorldPop population dataset [45] and water and sanitation points (e.g., boreholes and pit latrines) [46] to identify eight communities affected by flooding. These communities were confirmed by the National Disaster Management Organisation’s (NADMO) district office to be the communities most affected by dam release flooding.

Prior interactions (stakeholder meetings, site visits to flooded communities, and community mapping) took place with the communities, enhancing familiarisation with the flooding situation. A maximum variation sampling technique was employed in selecting respondents for the Focus Group Discussions (FGDs). Inhabitants of the eight flood-affected communities were recruited in person by referral from the local NADMO officer and the assembly members (a local elected representative), who were the point of contact. All respondents were 18 years and above old, had lived in the area for at least ten years, and had first-hand knowledge of floods in these localities. Participants were either district assembly members or chiefs, community opinion/influential leaders, or household heads. Only invited participants were present, and no one refused to take part. For the (KIIs), a purposive homogenous sampling strategy was used in selecting participants because of their community representatives (community representatives and chiefs or Traditional authority figures) and professionals with relevant roles in flood-related institutions (the health sector, local government or other organisations with a disaster relief remit) were purposefully selected to provide a range of perspectives (see Table 1). None of these selected participants declined participation.

**Table 1:** Characteristics of focus group discussion (FGD) and key informant interview (KII) participants in Savelugu and Talensi districts, Ghana.

District	Activity	Male	Female	Total
Savelugu	FGD	9	8	17
	KII	4	2	6
Talensi	FGD	8	8	16
	KII	5	1	6
**Total**		**26**	**19**	**45**

### Data collection

Data collection took place for two weeks, 14th– 27th September 2020, during the flooding period culminating in the Bagre dam release. All interviews and discussions were audio-recorded following a written informed consent of all participants. Both FDGs and KIIs were assisted by MA and FTG (Ghanaian male & female researchers, respectively, located outside the White Volta catchment). MA has a master’s degree in environmental geography, training in qualitative research methodologies, and six years of qualitative interviewing experience. FTG is a PhD researcher with a psychology and social science implementation background. She is a qualitative researcher with over seven years of experience.

Attendees for the FGD were flood-prone residents the facilitator had never met before. A participant information document containing the study goal and objectives was read to FGD and KII participants in a language they understood. One of the researchers took field notes during all conversations, while the other led the interaction. The FGD and KII tool were not piloted, and interviews were not repeated.

**FGDs**: Participants were invited to discussion sessions at a convenient location (Municipal Assembly conference hall), separating male from female participants to allow more open contributions on community and individual viewpoints and gender-sensitive issues [47]. A table of pseudonyms was created for each participant to record their comments. The mode of communication was in the local dialect (Dagbani and Frafra) and was translated by a professional translator whose gender matched that of the group concerned. Discussion groups comprised nine and eight participants (see Table 1), enabling each participant to contribute [48]. Topics discussed focused on the impacts of flooding on a community’s livelihood, life course, social, health, and economics, as well as short and long-term impacts (*see Online Resource 1*). A discussion session (face-to-face) lasted for about an hour and a half. On the fourth FGD, the topic had achieved saturation.

**KIIs:** Participants were interviewed in their offices and homes in English. A face-to-face 45-minute interview with semi-structured and open-ended questions focused on flooding’s impact on flood-related institution sectors and observations over time and challenges in delivering service to affected populations.

### Ethical approval and consent to participate

The study received ethical approval from the Ethics Committee of the Faculty of Environmental and Life Sciences, University of Southampton, U.K (Ref No: 54506, Approval date: 9^th^ February, 2020). and also from the Noguchi Memorial Institute for Medical Research, University of Ghana Ethics Review Committee (NMIMR-IRB CPN 062/19-20, Approval date: 4th March 2020). The ethics certificates were obtained for a bigger study titled “An assessment of flooding from dam releases and its impacts on diarrrrhoeal dissease and micibiological contamination of water sources in selected dryland areas of Northern Ghana”. The current manuscript emanated from this bigger study. To seek consent from study participants, a participant information was first read and explained in the local dialect to them through a translator and a written consent was sought thereafter.

### Data analysis

A hermeneutic strategy was used to analyse participants’ lived experiences of the flooding phenomenon [35]. Data were analysed inductively and deductively using thematic analysis approaches [49]. At the Immersion stage, MA and FTG repeatedly listened and transcribed all the recorded interviews verbatim, comparing results for omissions and accuracy. The transcripts were then imported into NVivo 12 software [50] during the understanding stage to identify codes (first-order participants construct). Based on the research questions and knowledge obtained from reading transcripts, a codebook was developed that guided the coding of all the data. All the transcripts were read line by line for coding. Additional codes were added to capture novel information emerging from the data set. The Abstraction stage identified sub-themes (second-order researcher construction). Sub-themes were grouped into themes and linked to sub-sector groups at the synthesis and theme development stage. There was also re-coding until the final themes and sub-themes were obtained (Table 2). There was triangulation across KII and FGD. Since these two methods provided an in-depth account of lived experience and observations, similar topics comparison, follow-ups and feedback after communicating results to participants were used to check for the reliability and validity of responses. Final themes and codes were exported back into Microsoft Word for further reading to inform interpretation. Themes and sub-themes were then cross-referenced to World Bank guidelines for conducting DaLA (Table 2), identifying whether each theme or sub-theme was captured through DaLA at the illumination and illustration of hermeneutic phenomena analysis circle stage. Themes were also classified as having immediate or delayed onset.

**Table 2:** Themes and sub-themes emanating from data analysis of focus group discussions with flood-affected community members, cross-referenced against Damage and Loss Assessment categories (World Bank, 2010).

Sector	DaLA category	Main theme	Sub-theme	Timing of onset
Agriculture and fishing	Productive sector: agriculture	Destruction of Agricultural produce	i. Interruption in Crop production ii. Damage to farming and fishing Assets	Immediate
Housing	Social sector: housing	Damage to buildings and loss of homes.	i. Ground saturation ii. Short-term: Inundation iii. Collapse	Immediate
Education	Social sector: education (i. only)	Interruption in teaching and learning activities	i. School attendance ii. School drop-out iii. Educational attainment	Immediate Delayed delayed
Health	Social sector: health	Potential health challenges and fatalities	i. High risk of danger ii. Experience of illness, injury or disease iii. Occurrence of deaths in a population. iv. Psychological consequences	Immediate Usually immediate Usually immediate Often delayed
Transport	Infrastructure sector: transportation	Road damage impeding mobility.	i. Reduced access to the food market. ii. Reduced access to healthcare	Immediate
Water and sanitation	Infrastructure sector: water and sanitation	Disruption of water and sanitation service	i. Contamination of water sources ii. Damage to the sanitation system.	Immediate
Ecology and Wildlife Conservation	n/a	Human-wildlife conflict	i. Intrusion in homes ii. Struggle for safe havens iiii. Attack on humans	Immediate
Social events and Welfare	n/a	Social events	i. Interruption of social engagements	Immediate
	n/a	Marriage, relationships, and child welfare	i. Decline marriage proposals ii. Child marriage iii. Divorce iv. Child welfare	Delayed

Findings were communicated to disaster management stakeholders, health experts, local government officials, and community residents, including participants, for comment and input during dissemination meetings in March 2022.

## Results

We present the results using typical categories in the World Bank’s Damage and Loss Assessment Framework (DaLA) [12]. First, we present impacts that would be captured through DaLA, then immediate impacts not usually captured through DaLA, and then delayed onset impacts not usually captured through DaLA, particularly those affecting the subsequent life courses of affected individuals.

### Community-reported impacts captured through Damage and Loss Assessment Framework

#### Agriculture and fishing sector

Destruction of Agricultural produce.

#### Damage to farming and fishing assets

Given that flood-plain recession agriculture and fishing are major livelihoods for Talensi and Savelugu’s populations, many immediate impacts relate to these activities, which take place in flood-plains. For example, a fisherman described the loss of fishing and farming equipment:

“*… All our farming tools and fishing equipment like a fishing net, canon, and basket are all carried away by the floods since we leave all at the river shore. Sometimes not even a single is left for the whole community”* **(FGD, Participant 5, Male, Talensi).**

***Interruption in crop production and yield*.** As illustrated in Table 3, flooding impacts farming systems and food security in affected communities, shortening cropping periods, delaying cultivation and transplanting of seedlings, reducing yields, and increasing post-harvest food losses.

**Table 3:** Effect of dam release flooding on crop cultivation and production in Northern Ghana.

Crop or vegetable	Flooding effect	Quotation
**Rice, Maize, groundnut or yam**	Post-harvest food losses	*“Others also harvested their corn and hid them in the farm to dry*, *and the water came and washed all their produce away”* **(FGD, Participant 2, Male, Talensi).**
	Reduced yields; shorter cropping periods	*“We usually time ourselves to harvest early enough so that by the time the flooding comes*, *we can carry the foodstuff to higher grounds… but this year we didn’t have the opportunity to take out*.* *.* *.*nothing was taken from the farm*. *We cultivated yam*, *groundnuts*, *maize*, *and nothing was harvested”* **(FGD, Participant 4, Female, Savelugu).**
**Onion, Pepper**	Delayed cultivation and delayed transplanting of seedlings	*“This time of the season*, *we farm onions and pepper*, *but there is still water in the areas we farm*, *so it has affected us farming these crops”* **(KII, 3, Male, Talensi).**

#### Social sector–Housing

*Damage to buildings and loss of homes*. Three sub-themes were identified relating to housing damage from flooding experienced in all study communities: ground saturation, inundation of houses, and collapse of houses. Almost all community members expressed grave concern regarding destroying their homes, as did the different categories of key informants (District/Municipal Chief Executives, NADMO officials, Chiefs, Assembly members, etc.). A participant lamented:

“*Now we are not even worried about our farm produce or the things we have on our farm, but rather we are struggling for our own home and houses where we sleep. The recent dam release and rains that came flooded our homes. In my compound, the level came up to my knees, and others to their waist and our room and dwelling places collapsed and are in very bad conditions because of the floods”* ***(FGD, Participant 2, Female, Savelugu).***

In some cases, entire communities were rendered homeless due to flooding, forcing residents to seek temporary shelter elsewhere and driving population displacement:

“…*In the Sugutampia community with about 39 houses, it was only left with four houses. It may even affect the remaining four houses as the water keeps coming in day by day”* **(KII, 2 Male, Savelugu).**

“*In our place*, *we are grouped into three communities*. *We have the Ewe*, *Hausa*, *and Mamprusi communities*. *As we speak*, *the Ewe community is left with three houses*. *The Mamprusi community is left with four houses*. *As for the Hausa*, *we’re not already plenty; all the structures have been pulled down by the floods”* **(Participant 5, FGD, Female, Savelugu).**

Saturated ground conditions caused building subsidence during or after flooding:

“*… the worse is, there are times that you even step foot in your room, and then, there’s a big hole created, and the water starts oozing. So, many of us in our rooms have water oozing out of our sleeping places right from the floor. The flooding has gotten so bad that the ground all over the place is wet and sinking. And any solid place is now covered with water, and even our cemented floors give way to water” (***Participant 3, FGD, Female, Savelugu***).*

#### Social sector–Education

*Interruption in teaching and learning activities*. FDG respondents identified three ways that flooding immediately affected children’s education: damage to school buildings; school buildings being used as temporary community shelters during floods; and children being unable to travel to school safely, as illustrated by the following comments:

“*We have a small river, a tributary of the White Volta here that sometimes makes it even more difficult for people to go to school. So, when there is a massive rainfall, we advise them not to cross and go to school*” (**FGD, Participant 1, Male, Talensi**).

*Our children cannot attend school because the buildings have collapsed or some are badly affected and are weak*. *We can not risk the lives of our children to go to school*. *Some communities are fortunate that their schools are not affected by the floods*, *but they are used as temporary shelters for the affected population*. *So*, *as long as the victims have occupied it*, *the children can not go to school (****KII*, *4 Male*, *Savelugu****)*.

#### Social sector–Health

*Potential health challenges and fatalities*. Flooding in northern Ghana has several health implications for individuals and communities in the affected areas. Flooding increases the risk of waterborne diseases. Flooding destroys crops, making it difficult for people to access food and leading to malnutrition, especially among children. Damages to homes and buildings lead to mold growth and dampness, which trigger respiratory problems such as asthma and allergies.

“*Water now occupies every inch of our communities. Our waste gets collected back into our water bodies. These are our sources for drinking and cleaning. So, when the flood comes, it contaminates our water sources, and we drink them. We know this, but we’re stuck and have no option.”* (**FGD, Participant 3, Female, Savelugu**).

Some other health conditions identified were consuming contaminated crops recovered after floods, bites from breeding mosquitoes, harsh weather conditions, and overcrowding in temporary flood shelters and settlements, leading to the spreading some infectious diseases. These effects of flooding affected all age groups and sexes. Health issues reported with exposure pathways (in descending order of frequency) included malaria as a result of the inability to set up bed nets in flood shelters; schistosomiasis (urinating blood) resulting from children playing in floodwaters; cholera; other diarrhoea; hookworm; respiratory infections and convulsions; and body sores, rashes, or skin diseases; accidents; and snake bites

“…*we are even exposed to sicknesses. The adults are not safe—much less the younger ones. The grounds are wet and are a source of sickness, even for the elderly and our kids. The place gets so wet that we are sometimes exposed to cold from outside. The normal thing is to get some warmth inside, but this time, it is the other way around. The cold starts from the inside”* (**FGD, Participant 4, Female, Savelugu**).

“*The children play with the flooded water a lot*, *so this causes bilharzia (urinating blood)”* (**FGD, Participant 3, Male, Talensi)**.

*“Now we’ve all converged on the community church to sleep*, *and there’s barely enough room to lay*, *let alone hang a mosquito net*. *So we’re all at risk*. *My child was just admitted to Diare Health Center*. *He has malaria*, *and you can hear my voice is shaky due to the cold*. *Overcrowding can also facilitate the spread of infectious diseases*.*”* **(FGD, Participant 6, Female, Savelugu**).

Flooding has reportedly claimed the lives of community members in various years from 1997 to 2019, though not in 2020.

“*A lot of people die through the Pwalugu river. In the past, in 1997, 1999, 2000, 2003, 2018, and 2019 there are records in the community to show this. In 2000, my sister died in a flooded river. All these years, people died, and everybody in my community is aware of this”* (**FGD, Participant 3, Male, Talensi)**.

“*In the past up to 2018*, *the effects of the flood have been bad*, *particularly the loss of human lives*. *In Talensi*, *in 2018*, *4 people died and a lot of hospital admissions due to the flooding”* (**KII, 5, Male, Talensi)**.

#### Infrastructure: Transport, water and sanitation

*Road damage impeding mobility*. Respondents reported how flood damage to roads and bridges seriously impedes mobility leading to access to food and healthcare outside of their communities. Particularly, communities selling agricultural produce, disrupt supply chains and cause food and medical supply shortages. Movement is restricted causing access to essential produce outside communities very cumbersome.

*“Some years back, the flood was so intense that the water stayed for 45 days before it receded, and it [the flood] divided the road into two. It was so flooded that vehicles could not cross from one side to the other”* ***(KII, 1, Male, Talensi).***

Another respondent similarly revealed that:

“*The flooding has been disturbing, and any time it comes, it destroys all our roads. When happens, we have people on the other side of the White Volta river, making it difficult for us to cross over there… We cannot even access the road to come out and buy food because every place is flooded, and we have been suffering all the time*” **(FGD, Participant 5, Male, Talensi).**

Most respondents expressed concern over the inaccessibility of communities during flooding. Health officials in both districts particularly recounted how their staff struggled to reach out to people in cut-off communities to provide medical care.

“*We do not hear much about the flooding apart from the roads being occupied with water. We are in the health sector, trying to reach every community in the district. In some communities, the health staff can’t access it, and they also can’t access the health facilities. Sometimes even referring emergency cases in a hospital from smaller health centres becomes problematic”* **(KII, 4, Female, Savelugu).**

Community members in both districts shared similar concerns over reduced healthcare access resulting from impassable roads during flooding. In some cases, this resulted in fatalities:

“*A woman gave birth at the district hospital. When she was discharged and going home, the baby fell in the water and died while the ‘motor king’ tricycle passed through the floods with the woman and the new baby”* **(FGD, Participant 1, Male, Talensi)**.

“*How to even get to the district hospital in times of emergency becomes an issue*. *During this time*, *people are cut off from the district hospitals*, *and some die as a result”* **(FGD, Participant 1, Male, Talensi).**

*Disruption of water and sanitation service*. Communities’ sanitation systems are also affected by flooding. During flooding, sanitation systems such as toilets and solid waste sites are affected.

“*The toilets facilities which serve the community are affected. Same can also be said for solid waste dumping sites. Some sometimes become inaccessible while in some instances, inundated water at the sanitation areas affects the nearby wells.”* **(FGD, Participant 3, Female, Savelugu).**

Finally, as reported elsewhere [31], FGD participants described how open defecation led to faecal contamination of water points during flooding. Microbiological contamination such as faecal enterococci, Shigella, and Salmonella was also reported to be present in the communities’ domestic water sources [28].

### Community-reported impacts with immediate onset not captured through DaLA

#### Human-wildlife contacts

Contact with dangerous wildlife was a theme that emerged from FGDs and KIIs but was absent from the DaLA guidelines. Flooding can displace both humans and animals, bringing people and wildlife into closer proximity as both compete for dry space and seek shelter. FGD respondents clearly found such incidents traumatic, as the following quotations exemplify:

“*In an attempt to drain the water coming into our home, which passes through our washroom into the compound, I made a guard at the entrance with buckets to fetch the water away. Then it trapped a snake, and I mistakenly picked it and threw it away without realising that it had collected a snake….”* **(FGD, Participant 5, Female, Savelugu).**

“*The situation is that we are even exposed to reptiles*. *Sometimes you are lying down*, *and sometimes you feel movement*. *Then you check*, *and you see a snake or a scorpion in your room”* **(FGD, Participant 3, Female, Savelugu).**

“*This area is home to snakes and scorpions*, *especially during this rainy season*. *Some snakes are even brought to this area by the dam release flood*. *The moving water sometimes carries wild animals from some distance to this area*.*”* (**KII, 1, Male, Talensi**).

During flooding, as people navigated flooded areas in canoes, there is a risk of exposure to reptiles:

“*Sometimes your canoe will pass under a tree, and a reptile (snakes or scorpions) will fall into your canoe from the tree because they also take shelter on the tree when the area is flooded. This wildlife will be alive and hungry because it has been there for some days without food. In this case, the fittest survive in the canoe or those who can swim faster”* **(KII, 1, Male, Savelugu)**

FGD respondents reported how flooding exacerbated risk of snake bite, with at least one observed case in each community:

“*Snakes bit two people in my community, and the same can be said for our neighbouring communities. We often experience snake bites*” **(FGD, Participant 4 and 7 Female, Savelugu).**

Despite efforts to have community members keep watch, the risk of snakebite remained high:

“*We put in place measures [during floods]. We don’t even sleep in our communities, but we are still exposed because reptiles are a major consideration why we don’t sleep and keep watch, but, upon all our measures, we still have people being victims of snake bites”* (**FGD, Participant 7, Female, Savelugu).**

#### Interruption of social engagements

KII respondents described flooding’s impact on community meetings, with religious services, weddings and other celebrations rescheduled, relocated, or held more quickly outdoors for fear of building collapse or repurposing of community buildings as shelters:

“*During this time, even when you call for a meeting, it will be difficult for people to attend because they may be cut off by inaccessible roads or fear entering a room with the fear of the building collapsing. Our usual meeting places might also be inundated with water***”. (KII, 1, Male Savelugu)**

“*People can’t go to people of worship because they are affected or used as temporary shelters for victims*. *One can not marry or do social events because nobody will come*.*”* **(KII, 2, Male Talensi)**

### Community-reported impacts with delayed onset not captured through Damage and Loss Assessment

#### Long-term mental health consequences

Flooding has long-term psychological consequences for mental health, particularly for those who have experienced significant loss or trauma due to flooding. People may experience Post-Traumatic Stress Disorder (PTSD), especially those who have lost loved ones or experienced significant property damage. This leads to symptoms of PTSD, including flashbacks, anxiety, and hypervigilance.

“*I can never forget how I lost my sister in 2000 in a flooded river.”* (**FGD, Participant 3, Male, Talensi)**.

Flooding can lead to feelings of uncertainty and anxiety about the future. Anxiety symptoms include excessive worry, panic attacks, and physical symptoms such as heart palpitations. Most of the FGDs and KII participants reported how the challenge of coping with all these effects traumatised them and often resulted in increased mental stress, anxiety, depression, and disease burden.

*“We are always stressed—most of the time, because of our fear of the unknown. Almost everybody here has someone sick at home. And some of us have been on medication as we are here. And sometimes, the psychological pressure from the stresses we must deal with all adds to our disease burden. One will not be in an appropriate state of mind knowing they must deal with these effects each year”. (* **FGD, Participant 4, Female, Savelugu** *).*

Flooding has ongoing stressors, such as financial challenges or difficulty finding a new home. This can lead to chronic stress, negatively impacting physical and mental health. Individuals may sometimes turn to drugs or alcohol to cope with the stress and trauma associated with flooding.

“*I know of one fine gentleman in my community who has abused drugs and alcohol due to losing his child [drowning]. His state is very pathetic. His wife also left him and has moved to the city.”* **(FGD, Participant 1, Female, Savelugu**).

#### Educational progression, attainment and school dropout risk

FGD participants described how flooding’s economic impacts had long-term consequences for children’s education. Families who have lost their homes or livelihoods may struggle to afford the cost of education, including school fees, uniforms, and supplies. This can force children to drop out of school and impact their educational attainment:

“*I lost everything during the flood. It affected my children going to school. They also lost their books, uniforms and other things. I had to pay their school fees and buy their books and uniforms, but everything [farm produce] I planted is gone and no money to feed, let alone pay school fees.They had to stay home this term”* **(FGD, Participant 1, male, Savelugu).**

Similarly, out-migration of married women searching for work as head-porters in cities during flooding affected the care and education of younger children, as acknowledged by all FGD and KII respondents, especially in polygamous households, where children mostly spend time with women. Some fathers do not pay attention to their children on school attendance. One woman reported:

“*When we leave home to do such work, on the other hand, it affects our children because they don’t get maximum care when with the father. It affects their school attendance”.* **(FGD, Participant 1, Female, Talensi).**

In many cases, floods can have long-term impacts on children’s education, particularly for those displaced or otherwise affected by the flooding. Female KII respondents reported children dropping out of school early, slower academic progression, and poor educational attainment:

*The challenge of school dropouts and poor performance in this district is seen or associated with flooding communities. Their student population reduces as they progress from one class to another. The few who can make it to the final are also not the best results. It is very worrying.* **(KII, 1, Female, Savelugu).**

Children who have experienced the trauma of a flood and the subsequent displacement may struggle with anxiety, depression, and other mental health and well-being challenges that can impact their academic performance and long-term outcomes

*We [family] moved here when I retired from work. When we were in the south, my children were doing well, but even since they experienced the flooding, they have been stressed and depressed. Their academic performance is on the decline. I regret moving here, but this place is our hometown.”* **(FGD, Participant 3, Male, Talensi)**

### Life course impacts: Marriage relationships, and child welfare

Three sub-themes emerged when we explored how flooding affected community members’ life courses: fewer successful marriage proposals, greater child marriages, and the out-migration of women seeking better livelihoods leading to divorce.

#### A decline in marriage proposal

Men living in flood-prone communities were reportedly less likely to propose marriage to women than men elsewhere successfully. A young man described how women’s fear of flooding prevented them from accepting marriage proposals from men residing in flood-prone communities, stigmatising those in flood-affected communities:

*“Sometimes, the flooding even affects our marriage. If a man around this place wants to marry, once the lady he has proposed to know he is from a flood-prone community like mine, she will turn the proposal down because she feels she won’t get anything. The flood will likely destroy all your properties, and her life is even in danger or at risk of such disaster” (* ***FGD, Participant 7, Male, Talensi*** *).*

*“It has sometimes become a stigma in the community*. *Once the community’s name is mentioned*, *the flooding is attached*. *Women and men around these communities sometimes experience rejection in marriage”*. *(***KII 1, Male, Talensi***)*

#### Early child marriage

Loss of livelihoods due to flooding and post-flood food insecurity compelled some families to offer their daughters for marriage at a younger age to secure dowry payments. Breast development of young girls served as the criteria in determining their readiness for marriage, as revealed by an older man:

*“Sometimes, we give our daughters in early marriage to get some money to feed the family. As low as 12 years old, that was the olden days, but now, we check the breast as soon as it is matured, so once we see some development in the breast, we can give them in marriage. She can become a fourth or fifth wife of a rich man; we don’t consider that a challenge”. (* ***FGD, Participant 2, Male, Talensi*** *).*

Some underage girls are also given to marriage to reduce their dependence on the family’s limited resources. The number of households reduces when girls are made to stay at their marital homes.

*“Sometimes we give them to marriage to make room for space and resources. For instance, if I have 3 girls and 3 boys, including myself and 2 wives, the whole family becomes 9. When the 3 girls leave, we are now left with 6. The harvest can last longer.”* (**FGD, Participant 4, Male, Savelugu)**

#### Out-migration, divorce, and child welfare

Female respondents reported the out-migration of married women from flood-prone communities particularly following the flood season and before the next farming season.:

*“What we [women] can do to help is that we have to travel and go down south (Kumasi, Techman, Accra) where there are a lot of commercial businesses needing labourer work (kayayi) to carry this on the head—[head porter], to earn some money for the next season’s farming….” (****FGD Participant 1, Female, Talensi***).

Male respondents recognised that out-migration and the financial impacts of flooding exacerbated underlying marriage relationships, increasing divorce risk. Despite leaving flood-prone communities intending to support their families by working elsewhere, flood-induced poverty meant some female migrants never returned home. Women’s prolonged absences sometimes resulted in the termination of existing marriages and women starting new relationships when they moved south for work. An elderly male respondent described how:

*“Sometimes they (married women) will travel to the cities (Kumasi) and go to do head-porter work (Kayaye). If they call to inquire about the flood and see that nothing has changed, some end up marrying there and not returning, leaving the family, including husband and children, behind”.* **(FGD, Participant 7, Male, Talensi).**

When a mother migrates during a flooding event, it can significantly impact the welfare and well-being of her children. Especially children do not get maximum care in a polygamous home. This sometimes causes malnutrition, retired growth, safety, and well-being.

*“It also affects their growth and health since sometimes they go on an empty stomach when times are hard” If the mothers are around, they will ensure the child eats, sleep well and has other basic needs. There are great differences between when a child’s mother travels and when she is around.”* **(FGD, Participant 1, Female, Talensi).**

## Discussion

### Life course and other impacts omitted from DaLA guidelines

Comparison of World Bank DaLA guidelines with community FGD and key informant interviews identifies flood impacts that would be omitted from a typical DaLA assessment, but which are an important part of the lived experience of flood-affected communities. These can be separated into immediate impacts and those with delayed onset, with the latter typically being subsequent life course events.

Among delayed onset impacts not captured by DaLA, our findings have shown that severe flood occurrence was associated with closely inter-related impacts including significant unemployment, income loss, and other indirect and long-term effects on life-course transition. Flooding exacerbated prevailing economic hardship and intensified out-migration, particularly of married women to southern Ghana, leading to reported higher divorce rates. This finding contradicts several studies in high-income countries, including a longitudinal and time series analysis in the USA [51], which reported stability and strengthening of romantic relationships following disasters. Similarly, Prati and Pietrantoni [52] noted an increase in the motivation of romantic partners to facilitate intimacy and partnership and maintain proximity to each other in the event of disasters in Italy. Furthermore, single men in flood-prone communities of Northern Ghana described how their marriage proposals were often unsuccessful. Women concerned about economic security through marriage tended to reject marriage proposals from males who were economically affected due to flood damage, a phenomenon also identified via longidutinal household survey analysis in Bangladesh [3].

In addition, flood-affected families sometimes arranged early marriages for their daughters, thereby receiving dowry money to counter lost income following flooding. Several respondents described using girls’ breast development as a traditional cultural indicator to determine readiness for an early marriage. Such socio-cultural context sexually objectifies females, equating their worth with their bodies’ appearance and sexual capabilities [53]. A high incidence of child marriage has previously been reported in northern Ghana [54], though this has not previously been connected to flood exposure. Early and polygamous marriages are culturally acceptable among the indigenous people of Northern Ghana. However, the practice endangers the girl child throughout her life course. Thus, despite changing social norms and government strategy supported by UNICEF [55], the girl child has not fully escaped the stigma of sexual objectification as flooding reinforces prevailing economic hardships and socio-cultural norms [56]. Ahonsi et al. [57] confirm the practice of child marriage in Northern Ghana and further explain its association with the poverty cycle for affected females. Consequently, flooding indirectly impedes the achievement of SDG 5, which aims to enhance girls’ education, promote gender equality, and eliminate child marriage in northern Ghana [58, 59]. Policymakers and researchers, therefore, need to target flood-affected communities in demystifying norms on the sexual objectification of young girls and apply appropriate sanctions to deter this practice.

Additionally, the out-migration of mothers from flood-prone communities may undermine their children’s subsequent welfare and education. The findings imply gaps in meeting children’s needs for optimal development. Female respondents reported male partners struggling to cope with parenting responsibilities in the absence of women, noting fathers’ limited ability to provide child care and the consequent effect on school attendance and child growth outcomes. Previous studies have highlighted women’s notable reproductive and productive roles in sustaining families [60] in Northern Ghana [61]. These role dynamics are explained by cultural factors that promote male superiority and discourage male engagement in household duties such as nurturing children [62].

Closely linked with the loss of livelihoods and the consequential out-migration of flood-affected community members are urbanisation and its associated challenges. These include ‘streetism’ for head porters and their children, high risk of disease spread, injury due to road accidents, exposure or involvement in prostitution, fraud, and petty crime [63]. Since perennial flooding in northern Ghana drives urbanisation [64], government and non-governmental organisations could intervene in rural flood-affected areas to reduce out-migration. Rakib *et al*. [65] identified skills acquisition and financial support as alternative measures that can enhance the capacity of affected households to recover from flooding and break the vicious cycle of poverty in disaster-prone locations.

Our respondents also reported immediate flood impacts not captured in DaLA guidelines. In particular, traumatic contact with dangerous wildlife was reported during flooding, with the associated risk of snakebite and other injuries, a risk not explicitly captured in DaLA guidelines [12]. The greater conflict between humans and wildlife has been attributed to processes that alter resource use for both populations, bringing them into competition and closer proximity [66]. Our study suggests flooding exacerbates this process as wildlife and humans compete for dry space and potable water. Respondents clearly found such incidents traumatic, so wildlife contact during flooding may constitute a specific risk factor for post-traumatic stress disorder in this setting, which is known to affect some of those exposed to flooding [67].

Finally, respondents reported other immediate impacts captured by DaLA guidelines and widely reported elsewhere. For example, acute health challenges such as access to healthcare facilities and increased infectious disease have been widely reported [68–70], as has loss of housing, agricultural, and fishing livelihoods and assets in flood-prone communities in other developing countries [17, 71, 72]. Significantly, dam release flooding in Northern Ghana affected the cultivation of onion and pepper and the harvesting of yam, groundnut, maise, and rice, undermining food availability among the rural poor. Although the dam operators, through NADMO, publicised schedules for spillovers, this warning was insufficiently early to allow farmers time to harvest some crops while other crops were not mature enough to harvest. Therefore, public and private investment should promote flood-resilient farming practices that ensure early readiness and harvesting of crops before the onset of floods [73].

### Limitations

Our findings are subject to some limitations. Although Focus Group Discussions (FGDs) were conducted in the local dialect and translated into English by a professional translator, some nuances could have been lost during translation. The study engaged resident household heads and opinion leaders; their perspectives could differ from those of the wider community. In particular, the perspectives of out-migrants or young girls could have differed greatly from those residents interviewed. Interviews took place following very extreme flooding, which could have magnified respondents’ accounts of flood impacts.

### Recommendations

These findings reinforce the need to incorporate community consultation into DaLA and post-disaster needs assessment to address longer-term flood impacts. Policy should support affected community members through flood-impacted life transitions, economic and financial adversity, and psychological trauma, since previous conventional interventions largely focus on short-term relief for individuals and communities. In particular, longer-term psychological and mental health support is recommended for flood-affected communities.

In the White Volta catchment, since respondents reported how stable marriages and parental relationships facilitated children’s health and educational development. Thus, to adequately compensate and rehabilitate flood-affected populations, policymakers and humanitarian relief providers should consider life course transitions and other physical or mental health problems, at least during the first year after the event. To transform these neighbourhoods, local, community, religious, and civil society agents resilient to such flood impacts should be incorporated into the rehabilitation process [3]. We also recommend investing in long-term flood prevention measures, such as building a holding dam below the Bagre Dam to hold flood water for other purposes, alongside soft loans for flood-suitable agricultural inputs or crops and targeted seasonal hardship funds to protect affected individuals. Such financial support, alongside life skills acquisition, could enable affected households to recover from the adverse effects of the flood and break the poverty cycle in such downstream flood-prone communities. Given the reported links between flood exposure and child marriage in our study, we recommend that government bodies, particularly Ghana’s Social Welfare Department and National Commission for Civic Education target existing educational programmes to reduce early child marriage [55] in flood-affected areas. Policymakers and researchers need to advance knowledge and practice, demystify norms on the sexual objectification of young girls, regardless of economic needs, and apply appropriate sanctions to deter such practices in flood-affected areas.

Future research should examine the influence of flood regimes on other life course transitions and their socioeconomic consequences (e.g., for educational, health, and employment outcomes) relative to non-flood-prone settings via a natural experimental, longitudinal study design.

### Conclusions

Although there are substantial benefits from dams that benefit multiple SDG targets, where climate change has led to greater rainfall intensity, their overspill can intensify the social, health, and environmental consequences of flooding for downstream populations.

In northern Ghana, participants reported how flooding decreased men’s marriage chances and reduced marital ties between individuals in non-flooded and flood-prone areas. Participants also described how some parents were compelled towards child marriage of girls to wealthy older men, to raise dowry funds to counter post-flood food insecurity and enable crop preparation for the following season. Flood events further destabilise adults’ marital relationships in flooded communities and lead to divorce and childcare challenges. Mothers who leave home during flooding to search for employment and higher living standards in cities will most likely not return. Children left behind by mothers most often drop out of school and suffer malnutrition. Flooding events in these communities interrupt social engagements, weakening social ties, school attendance and performance, churches and public gatherings are used as temporary shelters for affected households. Acute (short-term) health impacts and trauma from flooding reportedly lead to long-term mental health issues and trauma.

Disaster management, particularly DaLA guidelines, often neglects the long-term consequences of flooding and the recovery phase. Beyond its immediate consequences, long-term educational, societal, and mental health and trauma impacts of flooding on the life course may be neglected. These require addressing as much as short-term impacts, and these less tangible, long-term impacts further strengthen the case for preventing and mitigating flood impacts.
